# An unusual case of Lemierre syndrome – One pathogen or two?

**DOI:** 10.1016/j.idcr.2021.e01203

**Published:** 2021-06-21

**Authors:** Michael O’Shaughnessy, Dariel Irizarry, Diana Finkel

**Affiliations:** a Department of Medicine, Rutgers New Jersey Medical School, 185 S Orange Ave, Newark, NJ, 07103, United States; b Division of Infectious Diseases, Department of Medicine, Rutgers New Jersey Medical School, 185 S Orange Ave, Newark, NJ, 07103, United States

**Keywords:** Lemierre syndrome, *Fusobacterium necrophorum*, Group C streptococcus, Internal jugular vein thrombophlebitis

## Abstract

Lemierre syndrome, or septic thrombophlebitis of the internal jugular vein, is a rare disease that affects healthy young adults following an episode of pharyngitis or other upper respiratory disease. It most commonly involves the anaerobe *Fusobacterium necrophorum*, a component of normal oral flora. In this report, we present an unusual case of polymicrobial Lemierre syndrome involving both *F. necrophorum* and Group C streptococcus following an episode of pharyngitis and streptococcal toxic shock syndrome. Providers should consider the possibility of polymicrobial infection when there are imaging findings suggestive of Lemierre Syndrome and adjust antibiotic regimens accordingly.

## Highlights

• Lemierre Syndrome is characterized by thrombophlebitis of the internal jugular vein.
• The disease is rare, typically preceded by pharyngitis, and is caused usually by *Fusobacterium necrophorum*.
• In rare cases, Lemierre Syndrome is polymicrobial involving other oral microbes.

## Introduction

Lemierre Syndrome is a rare condition characterized by septic thrombophlebitis of the internal jugular vein, often leading to bacteremia and septic emboli. It is usually caused by the anaerobic gram-negative bacillus *Fusobacterium necrophorum*, a member of normal oropharyngeal flora [1]. Lemierre syndrome is most commonly preceded by an oropharyngeal infection such as pharyngitis, and presents with exudative tonsillitis, fever, sore throat, unilateral neck pain and tenderness, and respiratory symptoms if septic emboli are present in the lungs [2]. The disease typically affects otherwise healthy young adults [3]. We present the case of a healthy teenager who presented initially with Group C streptococcal pharyngitis and toxic shock syndrome requiring intensive care unit (ICU) admission and vasopressors who was later found to suffer from polymicrobial Lemierre Syndrome.

## Case report

The patient is a 20-year-old man with no significant past medical history who presented to the emergency department with an 8-day history of systemic complaints. He had initially presented to an outside hospital 4 days prior with similar complaints and was treated for a presumed viral infection. He reported sore throat, chest pain, episodes of non-bilious, non-bloody emesis, and diarrhea. He lived at home and denied sick contacts or recent dental work.

**Fig. 1:**
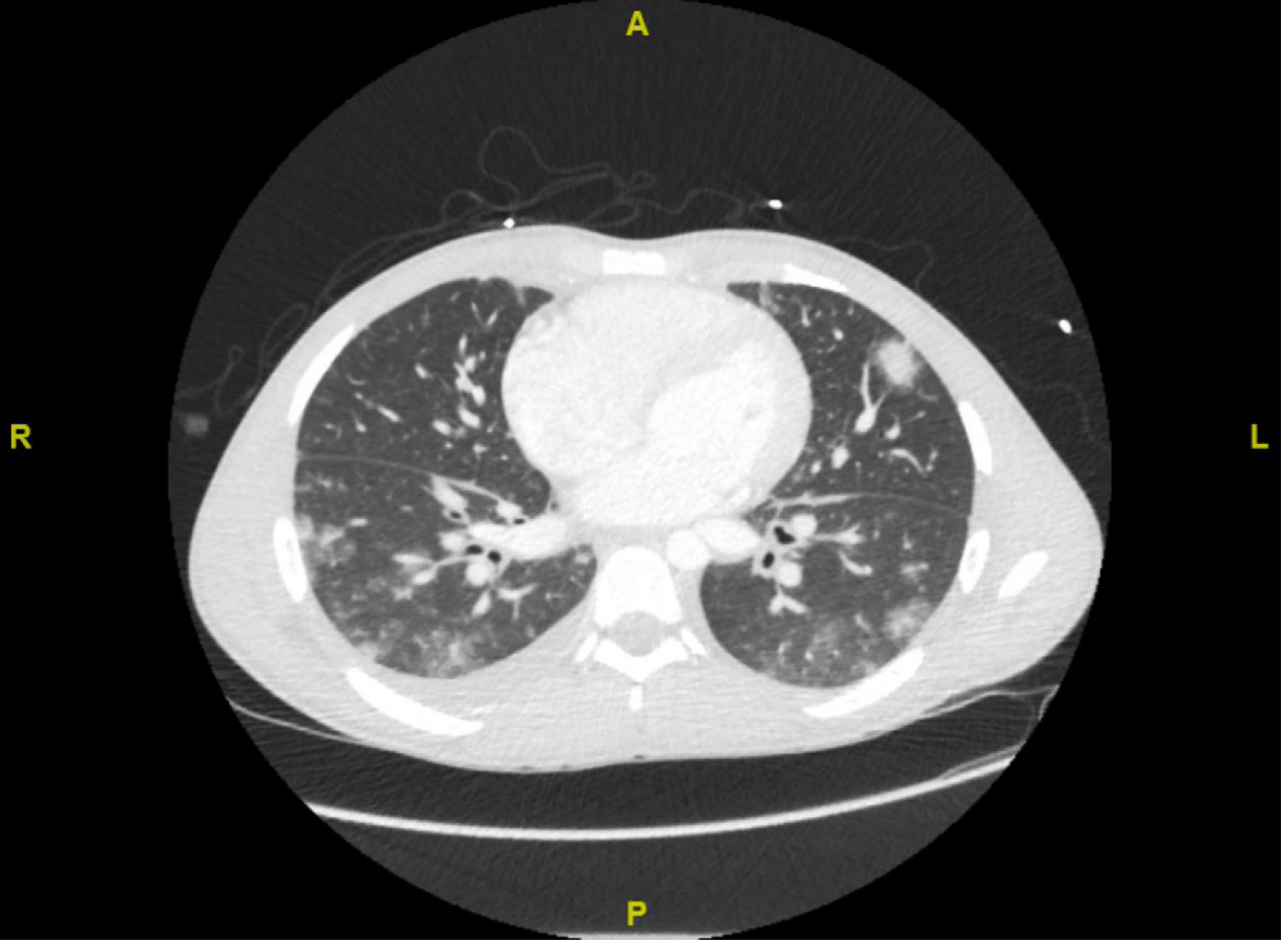
CT scan of the chest from Day 2 of the 1st hospitalization, showing diffuse ground-glass attenuation and consolidation of the lungs with lower lobe predominance.

In the emergency department (ED) the patient was febrile and hypotensive with systolic pressures in the 70 s–80 s mm Hg that did not respond to fluid resuscitation. Physical exam was pertinent for hepatosplenomegaly and tenderness over the right upper quadrant; no rash was present. Labs can be found in Table 1. He was pan-cultured (blood cultures at this time included only an aerobic bottle; anaerobic bottle was collected on day 3 of hospitalization) and was started on vancomycin, cefepime, and vasopressors. Computed tomography (CT) scan of the chest showed diffuse ground-glass attenuation and consolidation in both lungs consistent with multifocal pneumonia (Fig. 1) and a transthoracic echocardiogram (TTE) showed ejection fraction of 51 % and no vegetations. Septic emboli were considered less likely due to lack of cavitation.

**Table 1:** Lab values from both of the patient’s admissions; reference ranges based on institutional guidelines.

	Reference Range	Day 1 of 1^st^admission	Day 7 of 1^st^admission	Day 1 of 2^nd^admission	Day 5 of 2^nd^admission
WBC	4.0−11.0 × 10^3^/L	37.0 (72 % neutrophils, 15 % bands, 2% lymphocytes)	11.7 (66 % neutrophils, 1% bands, 17 % lymphocytes)	17.8 (86.8 % neutrophils, 5.3 % lymphocytes)	5.2 (31 % neutrophils, 40 % lymphocytes)
HGB	14.0−18.0 g/dl	13.2	9.8	10.2	9.0
Na	133−145 meq/L	129	133	126	132
K	3.7−4.8 meq/L	3.5	4.6	4.8	4.1
BUN	6−20 mg/dl	33	11	19	21
Cr	0.7−1.2 mg/dl	3.3	0.7	1.0	0.8
AST	0−40 U/L	58	25	82	27
ALT	0−41 U/L	33	19	72	38
Total Bili	<1.0 mg/dl	4.7 (3.6 direct)	1.6	0.9	0.5
Lactic Acid	0.5−2.2 mmol/L	4.7	n/a	0.7	n/a
D-Dimer	90−500 ng/mL	2,843	n/a	3,830	n/a
ESR	0−15 mm/hr	24	n/a	104	n/a
CRP	0−5 mg/L	236	n/a	65	n/a
Fibrinogen	145−490 mg/dl	496	n/a	n/a	n/a
LDH	120−250 u/L	303	n/a	n/a	n/a
Ferritin	30−400 ng/mL	1,288	n/a	n/a	n/a
Procalcitonin	<0.5 ng/mL	472	n/a	n/a	n/a
COVID-19 PCR	n/a	Negative	n/a	Negative	n/a
Rapid Strep	n/a	Negative	n/a	n/a	n/a

Infectious disease was consulted to assist in ascertaining the etiology of this pneumonia. Preliminary blood cultures grew gram-positive cocci in chains. Antimicrobials were adjusted to ceftriaxone, vancomycin, and clindamycin added to inhibit toxin production due to suspicion for streptococcal toxic shock syndrome.

In the following days the patient’s blood pressure and leukocytosis improved. (Table 1). Throat and aerobic blood cultures grew beta-hemolytic group C streptococcus and vancomycin was discontinued. Repeat blood cultures were negative, and the patient defervesced. Due to the patient’s initial presentation of group C streptococcus throat infection and septic shock due to toxic shock syndrome, the patient was directed to complete a 14-day course of intravenous (IV) ceftriaxone after discharge.

**Fig. 2:**
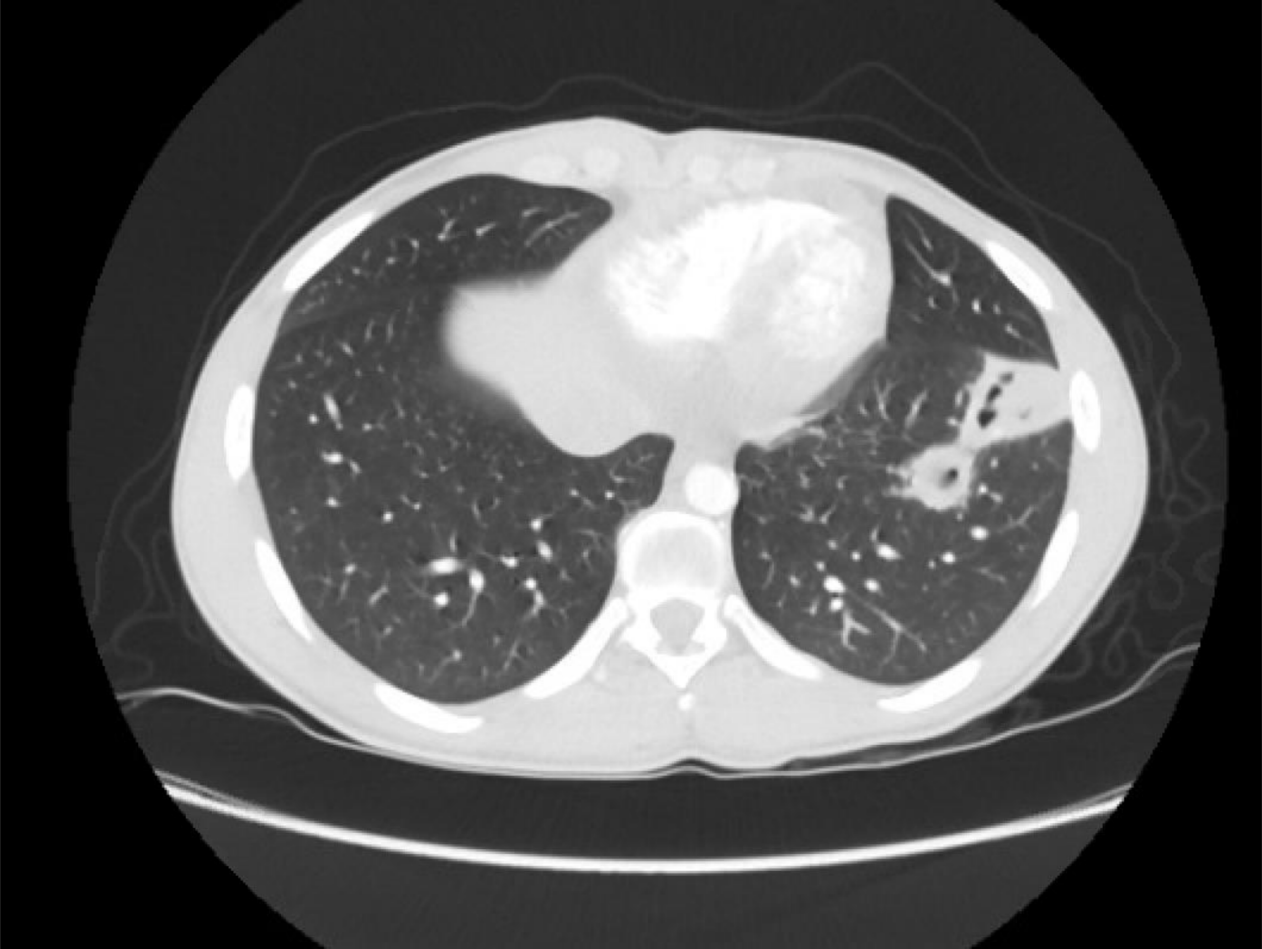
CT chest from Day 1 of 2nd admission showing airspace opacities with cavitation.

**Fig. 3:**
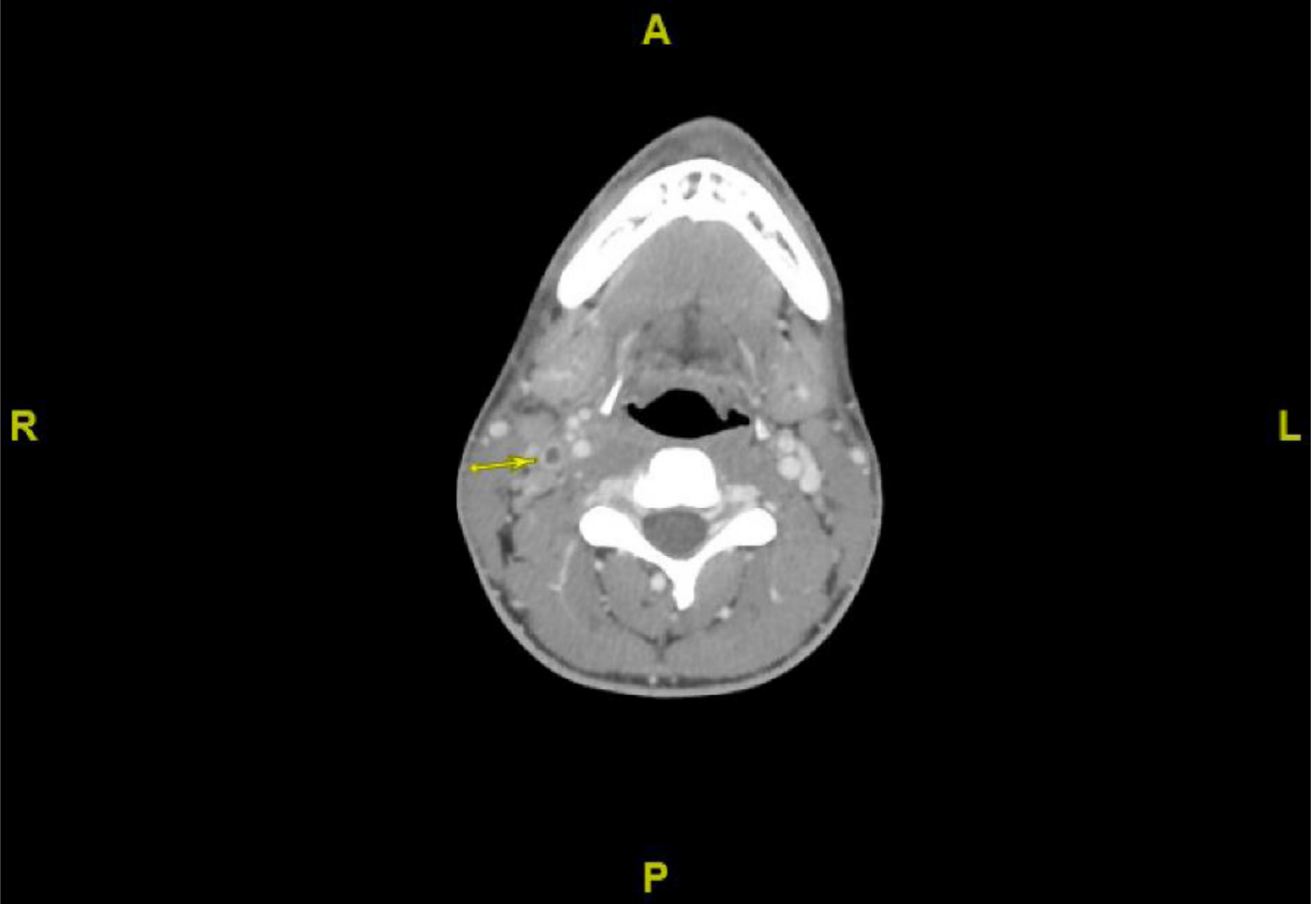
CT neck image from Day 1 of 2nd admission showing filling defect in R internal jugular vein (denoted by arrow), suggestive of septic thrombophlebitis and Lemierre Syndrome in the setting of *Fusobacterium necrophorum* bacteremia.

One day after discharge, final blood cultures from the hospitalization grew *Fusobacterium necrophorum* in the anaerobic bottle. Clinic follow-up was planned, but the patient returned to the ED four days after discharge due to new-onset fever, neck pain, and shortness of breath for one day. In the ED, he was febrile with otherwise stable vitals. Examination was significant for anterior neck tenderness. Labs can be found in Table 1. Repeat CT chest showed decreased opacities in the bilateral lungs and new cavitary lesions suspicious for septic emboli Figure 2 . CT neck revealed a filling defect in the right internal jugular vein (IJV) Figure 3 Metronidazole was added to the patient’s IV ceftriaxone for coverage of the *Fusobacterium*.

Anticoagulation was held as septic emboli were stable, and the patient defervesced with antimicrobial therapy. During admission, the patient remained afebrile and had improvement of his symptoms; his lab values also improved (Table 1). Blood cultures remained negative. The patient was discharged with a peripherally inserted central catheter (PICC) line to continue IV ceftriaxone and oral metronidazole for 4–6 weeks.

## Discussion

Lemierre syndrome is an exceedingly rare infection (estimated at 3.6 cases per million) involving septic thrombophlebitis of the internal jugular vein, typically with *Fusobacterium necrophorum,* following an oropharyngeal infection [1,3]. This patient fits this description, with the caveat that initial blood cultures grew beta-hemolytic Group C streptococcus in the setting of pharyngitis and septic shock. According to literature, while *Fusobacterium* alone is isolated from blood cultures in more than half of cases of Lemierre Syndrome, there are cases in which two organisms are implicated in Lemierre Syndrome, and other cases where blood cultures are negative [4,5]. A 2009 systematic review of case reports and case series involving Lemierre syndrome found that approximately 11 % of cases were polymicrobial, with less than 2% involving Group C streptococcus [5]. *Streptococcus* species have also been reported as the sole cause of IJV septic thrombophlebitis in rare cases. *Staphylococcus aureus* should also be considered as an etiology as it has been isolated in some cases, usually in cases involving jugular vein catheterization or preceding infections of skin or soft tissue [4,6,7]. This patient had no history of skin or soft tissue infection and did not receive an IJV catheter, so this was considered less likely.

Literature cites that the typical age of onset for Lemierre Syndrome is between 10 and 35 years old [8]. The etiology of septic thrombophlebitis caused by *Fusobacterium* is unclear due to the rarity of the disease; hypotheses include hematogenous spread through the tonsillar vein, lymphatic spread to the lateral pharyngeal space, and peritonsillar invasion [1]. It has also been suggested that primary bacterial or viral infection of the pharynx may alter the mucosal layer and allow for invasion of *Fusobacterium* [9].

Lemierre Syndrome is most frequently precipitated by pharyngitis, as in this case (although streptococcal toxic shock syndrome is an uncommon complication to precede the diagnosis Lemierre Syndrome); other less frequent causes include otitis, mastoiditis, and dental infections or abscesses [2,8]. The symptoms of sore throat, neck pain, and high fevers our patient presented with are typical of Lemierre syndrome; respiratory symptoms like dyspnea and pleuritic chest pain are common due to embolization to the lungs [1,2].

In the pre-antibiotic era, Lemierre syndrome had a case-fatality rate of up to 90 % [2,10]. Since the introduction of empiric antibiotic therapy, it has become much more treatable, with most mortalities coming due to delay in diagnosis. Current empiric antibiotic therapy options include piperacillin-tazobactam, carbapenems, or a regimen of ceftriaxone and metronidazole. Beta-lactamase resistant antibiotics are essential due to the isolation of resistant strains of *F. necrophorum* [11]. While this case demonstrates an uncommon presentation of polymicrobial Lemierre Syndrome, these empiric antibiotic therapies typically would cover for oral flora such as Group C streptococcus as well as *F. necrophorum.* The decision of whether to anticoagulate is controversial, as there is only anecdotal case report-based data suggesting that anticoagulation can prevent propagation of thrombus and septic embolization [12].

While the majority of cases of Lemierre Syndrome are caused by *F. necrophorum* alone, providers should keep in mind that Lemierre Syndrome can be caused by more than one pathogen at once, and antibiotic therapy may need to be tailored to other organisms in these cases.

## Author contribution

Michael O’Shaughnessy, B.S. – writing, care of patient.

Dariel Irizarry, M.D. – care of patient, writing, editing.

Diana Finkel, M.D. – care of patient, editing.

## Author statement

**Michael O’Shaughnessy:** Writing- original draft **Dariel Irizarry:** Conceptualization, Writing-review and editing **Diana Finkel:** Supervision, writing-review and editing.

## Consent

Written informed consent was obtained from the patient for publication of this case report and accompanying images. A copy of the written consent is available for review by the Editor-in-Chief of this journal on request.

## Ethical approval

Ethics committee approval was not obtained for this case report

## Funding

We have no sources of funding to declare.

## Declaration of Competing Interest

The authors report no declarations of interest.
